# Applying Theory to Understand and Modify Nurse Intention to Adhere to Recommendations regarding the Use of Filter Needles: An Intervention Mapping Approach

**DOI:** 10.1155/2014/356153

**Published:** 2014-07-10

**Authors:** Julianne Cassista, Julie Payne-Gagnon, Brigitte Martel, Marie-Pierre Gagnon

**Affiliations:** ^1^CHU de Québec, 2705 boulevard Laurier, Quebec City, QC, Canada G1V 2L9; ^2^Population Health and Optimal Health Practices, CHU de Québec Research Centre, 10 rue de l'Espinay, Quebec City, QC, Canada G1L 3L5; ^3^Nursing Directorate, CHU de Québec, 11 Côte du Palais, Quebec City, QC, Canada G1R 2J6; ^4^Faculty of Nursing, Université Laval, 2325 rue de l'Université, Quebec City, QC, Canada G1V 0A6

## Abstract

The manipulation of glass ampoules involves risk of particle contamination of parenteral medication, and the use of filter needles has often been recommended in order to reduce the number of particles in these solutions. This study aims to develop a theory-based intervention to increase nurse intention to use filter needles according to clinical guideline recommendations produced by a large university medical centre in Quebec (Canada). Using the Intervention Mapping framework, we first identified the psychosocial determinants of nurse intention to use filter needles according to these recommendations. Second, we developed and implemented an intervention targeting nurses from five care units in order to increase their intention to adhere to recommendations on the use of filter needles. We also assessed nurse satisfaction with the intervention. In total, 270 nurses received the intervention and 169 completed the posttest questionnaire. The two determinants of intention, that is, attitude and perceived behavioral control, were significantly higher after the intervention, but only perceived behavioral control remained a predictor of intention. In general, nurses were highly satisfied with the intervention. This study provides support for the use of Intervention Mapping to develop, implement, and evaluate theory-based interventions in order to improve healthcare professional adherence to clinical recommendations.

## 1. Introduction

Due to the properties of glass, glass ampoules are often used in the process of parenteral administration of medication. In fact, glass ampoules possess good chemical resistance; they are also impermeable and easy to clean and administer and can be sterilized and vacuumed. Moreover, glass ampoules allow for storage of photosensitive substances and the accurate measurement of medication [1]. However, the use of glass ampoules involves several risks, the most widely reported being the introduction of glass particles in medication [1–4]. The introduction of particles in parenteral injections can lead to complications for patients [5–8].

Filter needles can reduce or eliminate particles in medication administered parenterally [2, 4, 9]. Therefore, many authors and organizations have recommended the use of filter needles for parenteral injections [2–4, 9–16]. However, they are still not the norm in many settings [13], and nurses do not always adhere to clinical guidelines and recommendations regarding their use [14]. Furthermore, recommendations on the use of filter needles are not uniform [10–12, 17].

In a context where nurses are largely responsible for the preparation and handling of parenteral medication in hospital settings [6, 18–20], it is important to work on their perceptions since these perceptions can influence their willingness to follow current recommendations on the issue [21], including the use of filter needles in their practice.

**Figure 1:**
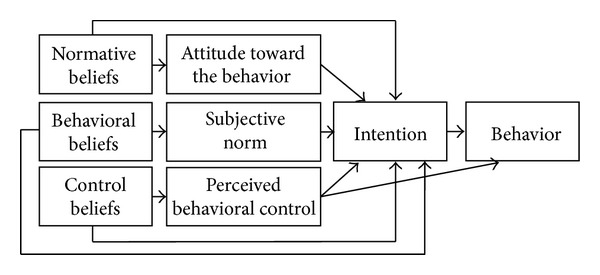
The theory of planned behavior (adapted from Ajzen, 1991) [22].

Ajzen's theory of planned behavior (TPB) [22, 23] is considered as a relevant theory to understand what influences the behaviors of healthcare professionals [24–26]. The TPB is based on the assumption that perceived behavioral control and intention are the two immediate determinants of behavior. Intention is in turn predicted by attitude, subjective norm, and perceived behavioral control. Each of these constructs is also influenced by specific beliefs that are, respectively, behavioral, normative, and control beliefs (see Figure 1). The TPB has been successfully applied in numerous healthcare settings [25] and could inform interventions aiming for behavioral modifications by identifying actionable factors [27].

In the first phase of this research project, conducted in a multisite university medical centre in the province of Quebec, Canada, we used the TPB to understand the determinants of nurse intention to follow recommendations on the use of filter needles in their practice. The results of this first phase are presented in another publication [30]. Based on these results, we developed, implemented, and evaluated an intervention based on the Intervention Mapping framework [31] in order to increase nurse intention to use filter needles according to clinical guideline recommendations. This paper presents the results of this intervention.

## 2. Methods

The Intervention Mapping (IM) framework was used to guide all the steps related to this intervention.

### 2.1. Intervention Mapping

IM is a framework elaborated by Bartholomew et al. [31] that provides a systematic approach for the development of interventions based on evidence and theory. IM consists of six steps: (1) needs assessments; (2) change in objectives; (3) theory-based methods and practical strategies; (4) program production; (5) adoption and implementation plan; and (6) evaluation plan. The first two steps were carried out during the first phase of the research project, which is published in another paper [30], but a summary is provided below. This part of the study focuses on steps three to six regarding the development, implementation, and evaluation of an intervention aimed at increasing nurse intention to follow recommendations on the use of filter needles in their practice.

### 2.2. Preliminary Steps

The two first steps of the IM framework were achieved through a collaborative project between the university hospital nursing directorate and a research team. Step 1, needs assessment, was based on a comprehensive assessment of the health problem, the program goals, and the desired changes in behavior. In the context of the release of new clinical practice guidelines regarding the use of filter needles in the organization, the nursing directorate identified a potential problem regarding nurse adherence to these guidelines, as it was seen as a key condition for the optimal use of filter needles. Due to the higher cost of the needles, their use was limited to specific circumstances, such as in the case of parenteral injection in newborns and children [17]. In collaboration with the research team, nurse leaders designed a research project to inform the implementation of the new recommendations regarding the use of filter needles in care units. Thus, the goal of the program was to ensure an optimal use of filter needles by nurses, informed by evidence-based recommendations. As these needles were made available in care units, individual nurse behavior was the only target for change; hence, this was an individual-level intervention.

For step 2, the behavior change objective was to improve nurse adherence to recommendations regarding the use of filter needles in their practice. The identification of behavioral determinants related to the change objective was achieved through a questionnaire survey among nurses from eight care units of the university medical centre. This survey identified attitude and perceived behavioral control, as well as three specific beliefs related to these constructs, as the main determinants of nurse intention to follow recommendations regarding the use of filter needles. The detailed results are presented elsewhere [30]. Thus, the performance objectives were informed by the two behavioral beliefs and the control belief identified from the survey. Performance objectives included, for instance, nurses explaining why using filter needles for parenteral injection in newborns and children according to recommendations is important; nurses demonstrating comfort using filter needles according to recommendations; and nurses acknowledging that using filter needles according to recommendations does not take more time.

### 2.3. Intervention

The third step of the IM framework consisted in the identification of strategies based on behavioral change theories. These change strategies were selected jointly by the nurse in charge of implementing guidelines on the use of filter needles (JC) and a researcher (MPG), using brainstorming and consulting the literature on behavior change strategies [31, 32]. We paid particular attention to the highly demanding context of practice of the targeted nurses and selected strategies that would take a minimum of their time. We also made sure to vary the types of strategies (passive and active, oral and written) and to encourage interaction.

**Figure 2:**
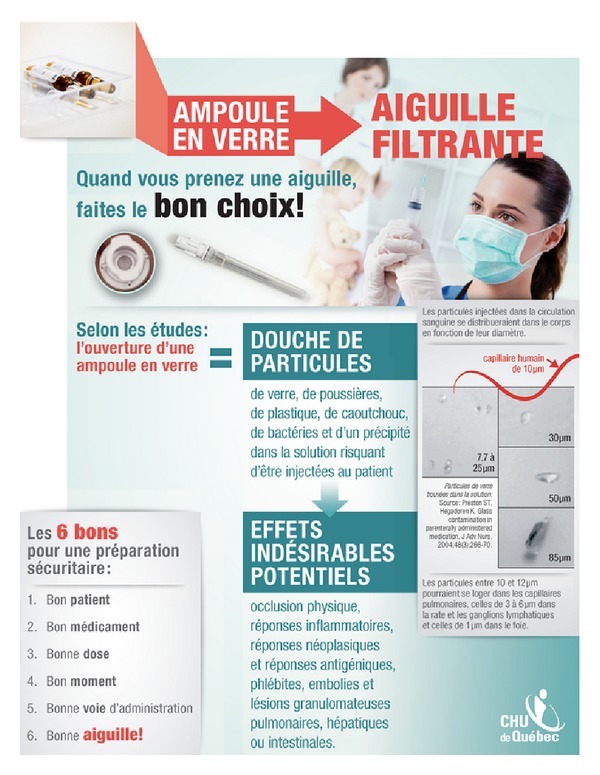
Poster for filter needles.

The fourth step of IM is the program production. This was again done jointly by JC and MPG, with input from the nursing directorate (BM), and included the creation of the program theme, its scope and sequence, and the list of needed materials. We designed the documents to guide the realization of the intervention by reviewing available materials and creating new ones if needed. For instance, a poster was designed based on a previous message about the “rights” of injection (see Figure 2). We made sure to follow theoretical considerations when specifying the parameters of behavior change methods (see examples in Table 1). Five nurses who were not from the targeted units validated the intervention program.

With respect to step 5 of the IM framework, program implementation was scheduled during the fall of 2013. All chief nurses from the target units were contacted beforehand and informed about the intervention. Two nurse trainers performed the intervention in five pediatric units of the university medical centre (three pediatric units, one neonatal intensive care unit, and one pediatric intensive care unit). In total, 36 sessions of 15 minutes in length were completed with the nurses working in these units between November 25 and 29, 2014 (*n* = 270).

The intervention included several components. First, the trainer presented scientific evidence on the negative effects of glass ampoules for patients and provided examples from the literature (e.g., dead infants that were fed parenterally in whom pulmonary artery granulomata were found [8]). Subsequently, information on research and recommendations about the use of filter needles was also presented to the nurses. Following this information, the trainer, who mastered the manipulation of filter needles, showed participants how to use them. While doing so, the trainer worked on the two constructs from the TPB that were identified as the main predictors of nurse intention in the first phase of the project, namely, attitude and perceived behavioral control (PBC). The specific beliefs targeted were enjoyment and reason (two behavioral beliefs) and ease of use (a control belief). After the demonstration by the trainer, two nurses were invited to do a simulation that compared the use of standard needles to the use of filter needles, and the whole group could point out the potential barriers to the use of filter needles on their units. Details regarding the steps of the simulation strategy are provided in Table 1.

After the intervention, posters were displayed in the area dedicated to the preparation of medication of each unit, informing about the potential risks related to particles from glass ampoules in medication and the six steps of a secure preparation: right patient, right medication, right dosing, right moment, right route of administration, and right needle (see Figure 2).

### 2.4. Evaluation of the Intervention

The sixth step of IM is the program evaluation. We used a before-after design with one group. Due to practical constraints, it was not possible to use a control group since the intervention units were not comparable to other care units in the hospital.

#### 2.4.1. Preintervention Questionnaire

In the first part of the project, self-administered questionnaires were distributed to all nurses (*n* = 364) of eight care units (five pediatric units and three adult intensive care units) in order to identify the primary determinants of nurse intention to follow recommendations related to the use of filter needles. The questionnaire was developed following experts' recommendations for the development of psychosocial questionnaires [33, 34]. Each construct from the TPB was measured by a certain number of questions (varying between three and six) and was assessed on a 7-point Likert scale.

#### 2.4.2. Postintervention Questionnaire

The postintervention questionnaire was developed based on results from the first phase of the study and pretested by four nurses who were not from the intervention units. It was distributed to all nurses from the five pediatric care units who participated in the intervention, just after its completion. As recommendations on the use of needles were limited to vulnerable groups, such as neonates and children, the intervention only concerned these five units. The postintervention questionnaire included two sections. The first section presented questions about the psychosocial determinants identified during the first phase of the study (six questions measuring attitude and three questions measuring PBC). Nurse intention to follow recommendations regarding the use of filter needles was assessed by a single item. The second section of the questionnaire assessed participants' satisfaction with the intervention based on six questions developed specifically for this study. All questions were assessed on a 7-point Likert scale. See Supplementary File 1 in Supplementary Material available online at http://dx.doi.org/10.1155/2014/356153 for the list of questions of the postintervention questionnaire (English translation—the original language being French).

Finally, the assistant head nurses of the various units were questioned about the use of filter needles one month after the intervention to learn if there were changes observed since the intervention.

### 2.5. Statistical Analyses

We conducted* t*-tests and Wilcoxon rank tests on all items and constructs to detect differences before and after the intervention. To test the theoretical model, we used a logistic regression in order to learn if attitude and PBC constructs had a significant influence on intention. We dichotomized the intention by classifying it in two categories: high intention (intention equal to 7) and moderate intention (intention below 7). Due to the very high postintervention intention score, the categories were unbalanced (intention of 7 = 162; intention below 7 = 6). Nurse satisfaction with the intervention was analyzed by conducting a descriptive analysis of the six items measuring it in the questionnaire. All statistical analyses were done using SAS 9.3 [35].

### 2.6. Ethical Considerations

Ethical approval was requested from the ethical committee of the university health centre. The committee concluded that approval was not necessary because the project was part of a quality improvement initiative. This study still adhered to the usual ethical considerations of informed consent, voluntary participation, and confidentiality.

## 3. Results

### 3.1. Participants

For the preintervention survey, 254 questionnaires were completed and returned on a possibility of 364 nurses working on the five pediatric units. Twelve questionnaires were excluded, resulting in 242 questionnaires included, for an effective response rate of 66.5%. For the postintervention questionnaire, 169 of the 270 nurses who received the intervention completed the questionnaire, for an effective response rate of 62.6%.  Table 2 presents participants' characteristics for the two questionnaires.

### 3.2. TPB Constructs before and after the Intervention

Since the theoretical constructs did not follow a normal distribution, the Wilcoxon rank test was deemed more appropriate than the* t*-test. However, both tests indicated that all variables were significantly higher after the intervention, as shown in Table 3.

A logistic regression model was used in order to learn whether the constructs targeted in the intervention (attitude and PBC) had a significant influence on intention. In the final regression model, only PBC remained as a predictor of intention, with an odds ratio of 3.60 (95% CI: 1.54–8.46; *P* = 0.0032). The final logistic regression model explained 32.5% (Nagelkerke *R* ^2^) of the variance in the intention score. The area under the ROC curve, which represents a measure of the correctness of the classification that would result from the regression model, was 0.74, which is considered as fair [36].

### 3.3. Satisfaction and Comments on the Intervention

The satisfaction questionnaire was developed in-house and comprised five specific items regarding the intervention and a global satisfaction item. We also computed the Cronbach alpha and performed a confirmatory factor analysis in order to verify the internal consistency of the items related to satisfaction from the postintervention questionnaire. These analyses showed good internal consistency (Cronbach *α* = 0.94) and confirmed that the items measured a single factor with an eigenvalue of 4.80.

The vast majority of nurses were highly satisfied with the intervention. Indeed, more than 95 percent of nurses responded 6 (agree) or 7 (totally agree) to the six questions related to satisfaction in the questionnaire (see Table 4 for details).

In addition, the follow-up with the assistant head nurses of the various units allowed us to gather additional comments regarding the effects of the intervention. In most cases, the assistant head nurses did not notice issues concerning the use of filter needles in their respective units, except in one unit that had problems obtaining needles during the first week. They also gathered comments from nurses who said they could not use standard needles anymore knowing what they could potentially inject into their patients. The assistant head nurses believed that the intervention convinced nurses to use filter needles with arguments based on research evidence.

## 4. Discussion

This study aimed to increase nurse intention to adhere to recommendations for clinical practice related to the use of filter needles, based on research evidence. Using the Intervention Mapping framework, a theory-based intervention was developed, implemented, and evaluated in five pediatric units of a large university medical centre in Quebec (Canada) in order to achieve this goal.

Nurse intention was already high in the preintervention questionnaire (mean of 6.69 on 7) and increased slightly after the intervention (mean of 6.94). This finding is important because, despite a high baseline value, it shows that the intervention could still improve nurse intention to follow clinical guideline recommendations. The same holds for the two theoretical constructs from the TPB that were targeted by the intervention. Both direct determinants of intention, that is, attitude and perceived behavioral control, significantly improved following the intervention. Moreover, all items measuring the beliefs associated with these constructs were significantly higher after the intervention. Of particular interest is the fact that the specific behavioral beliefs that were targeted by the intervention, namely, reason and enjoyment, showed a significant increase of 0.46 and 0.95, respectively. The control belief that was targeted by the intervention, ease of use, also showed an increase of 0.32 after the intervention.

PBC was the only construct that predicted nurse intention to follow recommendations related to the use of filter needles in their practice after the intervention, while attitude was not significant any longer. This could be due to the high intention scores among respondents, which left very limited variance to be explained. As PBC was the stronger determinant of intention prior to the intervention, this construct remained the only predictor in the postintervention regression.

This finding supports the fact that showing nurses how easy using filter needles can be increases the chances to improve their adherence to recommendations related to their use. Many studies have reported PBC as a prominent predictor of healthcare professional intention to follow clinical recommendations. Among them, the Kortteisto et al. study on physician intention to use guidelines [37], the Foy et al. study on adherence to guidelines in gynecology units in Scotland [38], and the Rashidian and Russell study on adoption of guidelines related to asthma by general practitioners [39] all concluded that perceived behavioral control was the most important predictor of intention.

It is worth noting that the vast majority of nurses seemed very satisfied with the intervention. As we were not aware of any tools available in French to assess nurse satisfaction with a behavior change intervention, we developed our own tool. We hope that this simple six-item questionnaire can be used for evaluating participants' satisfaction with other similar interventions.

Although it was not possible to assess nurse behavior in this study due to resource constraints, we did an informal follow-up with the assistant chief nurses of all five units that received the intervention. Apart from a problem regarding the availability of filter needles in one unit during the week following the intervention, all assistant chief nurses said that nurses were very keen to follow recommendations regarding the use of filter needles in parenteral injections. Nurses mentioned that they had no choice but to use filter needles because they were now aware, based on scientific evidence, of the potential harms to patients associated with the injection of glass particles.

### 4.1. Limitations

There are several limitations to consider in this study. First, care units that participated in this study come from a single multisite university medical centre in the province of Quebec (Canada). Although the results may be different in other settings and may consequently be difficult to generalize, we believe that it is possible to follow the approach for targeting similar behaviors in other settings of care since the study applies the Intervention Mapping framework [31], a structured approach to theory-based interventions. Second, it was impossible for us to match the answers of participants from the first phase of the study to those of the second phase because the questionnaires were entirely anonymous. This limited the possibility of applying more robust analyses to test the differences before and after the intervention. We had to consider the two samples as independent although they should have been treated as nonindependent. Third, we used a single group before-after design to assess the intervention's effects on nurse intention. The use of a more robust experimental design such as the randomized controlled trial or controlled before-after study was not possible due to limited resources and constraints related to this real-life intervention. In fact, only the five pediatric units in which the intervention was conducted were targeted by the decision to recommend the use of filter needles in parenteral injections in this university medical centre, limiting the possibility of having a control group.

## 5. Conclusion

Clinical recommendation adoption and adherence among healthcare professionals is a continuous challenge. This study, based on the Intervention Mapping framework and the theory of planned behavior, allowed for the development, implementation, and evaluation of a theory-based intervention among pediatric care nurses in order to improve their intention to follow clinical recommendations regarding their use of filter needles. Based on the results, we believe that the intervention did improve the intention of these nurses to use filter needles according to recommendations in their practice. In light of this study, theory-based interventions may be a potential solution to improve adherence of healthcare professionals to clinical recommendations.

## Supplementary Material

Postintervention questionnaire (translated from French).

## Figures and Tables

**Table 1:** Details of the simulation strategy.

Step (theoretical construct targeted)	Details	Parameters (examples)
(1) Presentation of scientific evidence (knowledge)	Present scientific evidence on the negative effects of glass ampoules for patients, information on research, and recommendations about the use of filter needles.	For modeling [28]: attention, remembrance, self-efficacy and skills, reinforcement of model, and identification with model.
(2) Preparing medication from a glass ampoule (perceived behavioral control)	Two nurses prepare medication contained in a glass ampoule (e.g., furosemide or narcotic). One uses a filter needle; the other uses a standard needle.	
(3) Positive feedback (reinforcement)	Do positive feedback during the simulation (ability of nurses to manipulate the equipment and to prepare medication).	For reinforcement [28]: reinforced by significant other.
(4) Pointing out ease of use (perceived behavioral control)	Point out the ease of use of filter needles.	
(5) Mental imagery of glass particles (attitude)	Lead nurses through imagining glass particles (with pictures) injected in the patient and the microorganisms that become lodged in the capillaries, blocking them.	
(6) Comparing time between filter and standard needles (perceived behavioral control)	When the medication is prepared, do a comparison between the time taken to prepare the medication with the filter needle and the standard needle.	For persuasion [29]: repeated connections between behavior and positive feelings.
(7) Verbalization of the situation	Allow time for verbalization of the situation for the nurses who prepared the medication.	
(8) Inversing the roles (perceived behavioral control)	Do the simulation again by inversing the roles of the two nurses.	
(9) Feedback and evaluation (perceived behavioral control)	Following the simulation, evaluate the potential barriers to the use of filter needles on the unit. Find solutions, if possible. Offer follow-up if there is an impasse or obstacle that was not resolved.	
(10) Verbalization of the intervention	Allow time for a verbalization of the overall experience with the intervention.	

**Table 2:** Characteristics of the participants, before and after intervention.

(1) Preintervention questionnaire		
Participants' characteristics	All participants	
	(*n* = 242 (239)^1^)	
	*n*	%
Care units		
1 (ICU)	18	7.4
2 (PICU)	32	13.2
3 (NICU)	56	23.1
4 (pediatric)	12	5.0
5 (pediatric)	39	16.1
6 (pediatric)	16	6.6
7 (ICU)	21	8.7
8 (ICU)	48	19.8
Gender		
Male	30	12.6
Female	209	87.4
Age		
19–25	52	21.7
26–30	52	21.8
31–35	36	15.1
36–40	28	11.7
>40	71	29.7
Highest educational grade		
College	99	41.4
Certificate	10	4.2
B.S.	123	51.5
M.S.	3	1.3
Other studies	4	1.7
Years of experience		
0–5	79	33.1
6–10	48	20.1
11–15	44	18.4
>20	57	23.8

(2) Postintervention questionnaire		
Participants' characteristics^2^	All participants	
	(*n* = 169)	
	*n*	%

Care units		
2 (PICU)	20	51.3
3 (NICU)	65	50.0
4 (pediatric)	25	83.3
5 (pediatric)	36	80.0
6 (pediatric)	23	88.5

^1^Three respondents did not answer the four questions related to sociodemographic characteristics (gender, age, education and experience), reducing the total number of participants for these questions to 239.

^2^Other sociodemographic data were not collected for this questionnaire.

**Table 3:** Differences between pre- and postintervention scores on survey items^1^.

Item	Mean score before intervention (SD)	Mean score after intervention (SD)	Difference	*P* value
ATT1	6.51 (0.88)	6.88 (0.46)	0.37	<0.0001
ATT2	6.24 (0.97)	6.85 (0.38)	0.61	<0.0001
ATT3	6.43 (0.96)	6.89 (0.54)	0.46	<0.0001
ATT4	5.95 (1.20)	6.79 (0.55)	0.85	<0.0001
ATT5	5.15 (1.28)	5.90 (1.32)	0.75	<0.0001
ATT6	5.05 (1.31)	6.00 (1.20)	0.95	<0.0001
PBC1	6.53 (0.72)	6.84 (0.70)	0.32	<0.0001
PBC2	6.31 (1.08)	6.68 (1.15)	0.36	0.0014
PBC3	6.65 (0.73)	6.93 (0.50)	0.28	<0.0001
INT1	6.69 (0.66)	6.94 (0.49)	0.25	<0.0001

^1^We present the *t*-test instead of the Wilcoxon rank test to facilitate understanding of the data. The two tests produced the same results.

**Table 4:** Nurse satisfaction with the intervention.

Item		*n* of responses below 6 (%^1^)	*n* of responses of 6 or 7 (%^1^)
*The training received concerning the use of filter needles… *			
SAT1	Met my needs	5 (3.0)	164 (97.0)
SAT2	Seemed pertinent to me	2 (1.2)	167 (98.8)
SAT3	Was of an adequate length	3 (1.8)	166 (98.3)
SAT4	Made me learn something	4 (2.4)	165 (97.7)
SAT5	Will be useful for my practice	2 (1.2)	167 (98.8)
SATG	In general, what is your level of satisfaction related to the training received?	5 (3.0)	163 (97.0)

^1^Percentage after exclusion of missing values.
