# Supplementary material for: Applying Theory to Understand and Modify Nurse Intention to Adhere to Recommendations regarding the Use of Filter Needles: An Intervention Mapping Approach
Source: Nurs Res Pract. 2014 Jul 10;2014:356153. doi: 10.1155/2014/356153 (PMC4121269; doi:10.1155/2014/356153)
Supplement: Supplementary file 1 — Postintervention questionnaire (translated from French). [file 356153.f1.docx]

| **For me, using filter needles according to the recommendations from the interactive care methods (ICM)^1^ would be:** | | | | | | | | | | | | | | | |
| --- | --- | --- | --- | --- | --- | --- | --- | --- | --- | --- | --- | --- | --- | --- | --- |
| **ATT1** | **1. *(Security)*** | | | | | | | | | | | | | | |
| 1. Very risky | | 2. Risky | | | | | | 3. Slightly risky | | 4. Neither | | 5. Slightly Safe | | 6. Safe | 7. Very safe |
| **ATT2** | **2. *(Complexity)*** | | | | | | | | | | | | | | |
| 1. Very complex | | 2. Complex | | | | | | 3. Slightly complex | | 4. Neither | | 5. Slightly simple | | 6. Simple | 7. Very simple |
| **ATT3** | **3. *(Reason)*** | | | | | | | | | | | | | | |
| 1. Very unreasonable | | 2. Unreasonable | | | | | | 3. Slightly unreasonable | | 4. Neither | | 5. Slightly reasonable | | 6. Reasonable | 7. Very reasonable |
| **ATT4** | **4. *(Satisfaction)*** | | | | | | | | | | | | | | |
| 1. Very unsatisfying | | 2. Unsatisfying | | | | | | 3. Slightly unsatisfying | | | 4. Neither | 5. Slightly satisfying | | 6. Satisfying | 7. Very satisfying |
| **ATT5** | **5. *(Reward)*** | | | | | | | | | | | | | | |
| 1. Very demeaning | | | 2. Demeaning | | | | | 3. Slightly demeaning | | | 4. Neither | 5. Slightly rewarding | | 6. Rewarding | 7. Very rewarding |
| **ATT6** | **6. *(Enjoyment)*** | | | | | | | | | | | | | | |
| 1. Very unpleasant | | | 2. Unpleasant | | | | | 3. Slightly unpleasant | | | 4. Neither | 5. Slightly pleasant | | 6. Pleasant | 7. Very pleasant |
| **PBC1** | **7. For me, it will be easy to use filter needles according to the recommendations from the ICM** | | | | | | | | | | | | | | |
| 1. Very unlikely | | | 2. Unlikely | | | | | 3. Slightly unlikely | | | 4. Neither | 5. Slightly likely | | 6. Likely | 7. Very likely |
| **PBC2** | **8. There won’t be obstacles to the use of filter needles according to the recommendations from the ICM** | | | | | | | | | | | | | | |
| 1. Very unlikely | | | | 2. Unlikely | | | | | 3. Slightly unlikely | | 4. Neither | 5. Slightly likely | | 6. Likely | 7. Very likely |
| **PBC3** | **9. I believe I am capable to use filter needles according to the recommendations from the ICM** | | | | | | | | | | | | | | |
| 1. Very unlikely | | | | 2. Unlikely | | | | | 3. Slightly unlikely | | 4. Neither | 5. Slightly likely | | 6. Likely | 7. Very likely |
| **INT1** | **10. I have the intention to use filter needles according to the recommendations from the ICM** | | | | | | | | | | | | | | |
| 1. Very unlikely | | | | | 2. Unlikely | | | | 3. Slightly unlikely | | 4. Neither | 5. Slightly likely | | 6. Likely | 7. Very likely |
| **On a seven-point scale, 1 signifying that you completely disagree and 7 that you completely agree, please circle your level of agreement with the following affirmations.**  **The training received concerning the use of filter needles…** | | | | | | | | | | | | | | | |
| ***SAT1*** | **11. Met my needs** | | | | | | | | | | | | | | |
| *1.* | | | | | 2. | | | | 3. | | 4. | 5. | | 6. | 7. |
| **SAT2** | **12. Seemed pertinent to me** | | | | | | | | | | | | | | |
| *1.* | | | | | | 2. | | | 3. | | 4. | 5. | | 6. | 7. |
| **SAT3** | **13. Was of an adequate length** | | | | | | | | | | | | | | |
| *1.* | | | | | | 2. | | | 3. | | 4. | 5. | | 6. | 7. |
| **SAT4** | **14. Made me learn something** | | | | | | | | | | | | | | |
| *1.* | | | | | | | 2. | | 3. | | 4. | 5. | | 6. | 7. |
| **SAT5** | **15. Will be useful for my practice** | | | | | | | | | | | | | | |
| *1.* | | | | | | | 2. | | 3. | | 4. | | 5. | 6. | 7. |
| **SATG** | **16. In general, what is your level of satisfaction related to the training received?** | | | | | | | | | | | | | | |
| *1.* | | | | | | | 2. | | 3. | | 4. | | 5. | 6. | 7. |

^1^ The interactive care methods (ICM) is a tool created by the Quebec University Health Center (CHU de Québec) and Solution Hospitalis. It consists of monographs on clinical recommendations, techniques, and procedures usually performed by nurses. It presents a step by step process to provide the care required to patients, using visual support.
